# Methyl 2-(8a-hy­droxy-4a-methyl-8-methyl­enedeca­hydro­naphthalen-2-yl)acrylate

**DOI:** 10.1107/S1600536811053712

**Published:** 2011-12-17

**Authors:** Mohamed Tebbaa, Ahmed Benharref, Abdelghani Oudahmane, Fouad Mellouki, Moha Berraho

**Affiliations:** a bLaboratoire de Chimie Biomoléculaire, Substances Naturelles et Réactivité, URAC 16, Faculté des Sciences Semlalia, BP 2390, Bd My Abdellah, 40000 Marrakech, Morocco; b Universite Blaise Pascal, Laboratoire des Mate’riaux Inorganiques, UMR CNRS 6002, 24 Avenue des Landais, 63177 Aubie‘re, France; c Laboratoire de Chimie Bioorganique et Analytique, URAC 22, BP 146, FSTM, Université Hassan II, Mohammedia-Casablanca 20810 Mohammedia, Morocco

## Abstract

The title compound, C_16_H_24_O_3_, was synthesized from ilicic acid which was isolated from the aerial part of *Inula Viscosa­* (L) Aiton [or *Dittrichia Viscosa­* (L) Greuter]. The mol­ecule contains two fused six-membered rings both in chair conformations. In the crystal, mol­ecules are linked into chains running parallel to the *a* axis by O—H⋯O hydrogen bonds.

## Related literature

For the synthesis, see: Barrero *et al.* (2009 ▶). For the medicinal and pharmacological properties of *Inula* Viscosa (L) Aiton [or *Dittrichia Viscosa­* (L) Greuter], see: Shtacher & Kasshman (1970 ▶); Bohlmann *et al.* (1977 ▶); Chiappini *et al.* (1982 ▶); Azoulay *et al.* (1986 ▶); Bohlmann *et al.* (1977 ▶); Ceccherelli *et al.* (1988 ▶). For background to phytochemical studies of plants, see: Geissman & Toribio (1967 ▶). For conformational analysis, see: Cremer & Pople (1975 ▶). 
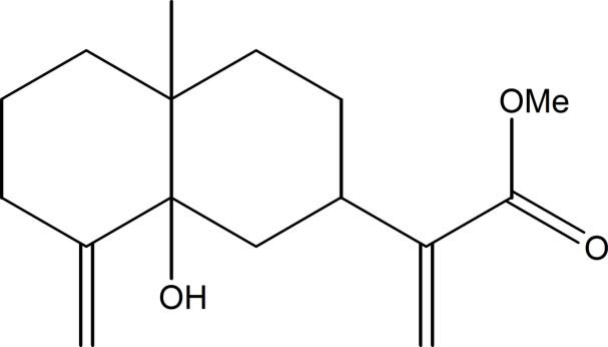


## Experimental

### 

#### Crystal data

C_16_H_24_O_3_
*M* *_r_* = 264.35
Orthorhombic, 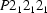

*a* = 6.0666 (5) Å
*b* = 10.0900 (9) Å
*c* = 23.747 (2) Å
*V* = 1453.6 (2) Å^3^
*Z* = 4
Mo *K*α radiation
μ = 0.08 mm^−1^
*T* = 296 K
0.65 × 0.45 × 0.26 mm

#### Data collection

Bruker APEXII CCD diffractometer
6831 measured reflections
1745 independent reflections
1202 reflections with *I* > 2σ(*I*)
*R*_int_ = 0.050

#### Refinement

*R*[*F* ^2^ > 2σ(*F* ^2^)] = 0.046
*wR*(*F* ^2^) = 0.114
*S* = 1.04
1745 reflections
176 parameters
H-atom parameters constrained
Δρ_max_ = 0.18 e Å^−3^
Δρ_min_ = −0.19 e Å^−3^

### 

Data collection: *APEX2* (Bruker, 2005 ▶); cell refinement: *SAINT* (Bruker, 2005 ▶); data reduction: *SAINT*; program(s) used to solve structure: *SHELXS97* (Sheldrick, 2008 ▶); program(s) used to refine structure: *SHELXL97* (Sheldrick, 2008 ▶); molecular graphics: *ORTEP-3 for Windows* (Farrugia, 1997 ▶) and *PLATON* (Spek, 2009 ▶); software used to prepare material for publication: *WinGX* (Farrugia, 1999 ▶).

## Supplementary Material

Crystal structure: contains datablock(s) I, global. DOI: 10.1107/S1600536811053712/bt5751sup1.cif

Structure factors: contains datablock(s) I. DOI: 10.1107/S1600536811053712/bt5751Isup2.hkl

Supplementary material file. DOI: 10.1107/S1600536811053712/bt5751Isup3.cml

Additional supplementary materials:  crystallographic information; 3D view; checkCIF report

## Figures and Tables

**Table 1:** Hydrogen-bond geometry (Å, °)

*D*—H⋯*A*	*D*—H	H⋯*A*	*D*⋯*A*	*D*—H⋯*A*
O1—H1⋯O3^i^	0.82	2.24	3.033 (4)	161

Symmetry code: (i) 
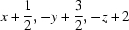
.

**Fig. 1.**
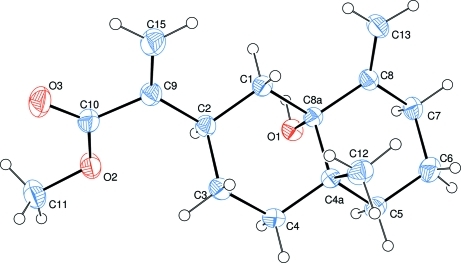
: Molecular structure of the title compound with the atom-labelling scheme. Displacement ellipsoids are drawn at the 30% probability level. H atoms are represented as small spheres of arbitrary radii.

**Fig. 2.**
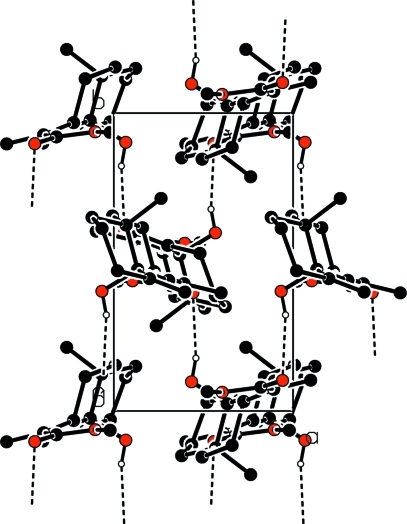
: Partial packing view showing the O–H···O interactions (dashed lines) and the formation of a chain parallel to the a axis. H atoms not involved in hydrogen bonding have been omitted for clarity.

## supplementary crystallographic information

### Comment

The Inula Viscosa (*L*) is widespread in Mediterranean area and extends to the Atlantic cost of Morocco. It is a well known medicinal plant (Shtacher & Kasshman, 1970; Chiappini *et al.*, 1982) and has some pharmacological activities (Azoulay *et al.*, 1986). This plant has been the subject of chemical investigation in terms of isolating sesquiterpene lactones (Bohlmann *et al.*, 1977), sesquiterpene acids (Ceccherelli *et al.*, 1988; Geissman & Toribio, 1967). The ilicic acid is one of the main components of the dichloromethane extract of the Inula Viscosa (*L*) Aiton or Dittrichia Viscosa (*L*) Greuter]. The literature report one article on the transformation of the ilicic acid (Barrero *et al.*, 2009). In order to prepare products with high added value, that can be used in the pharmacologycal industry, we have studied the reactivity of this sesquiterpene acid. Thus, from this acid, we have prepared by the method of Barrero *et al.* (2009), 2-(4a,8-Dimethyl-1, 2,3,4,4 a,5,6,7- octahydro naphthalen-2-yl)-acrylic acid methyl ester. The epoxidation of the latter compound by metachloroperbenzoic acid (mCPBA), followed by the opening of the epoxide obtained by Bi(OTf)_3_ leads to the title compound (I) with a yield of 50%.

The molecule is built up from two fused six-membered rings. The molecular structure of (Fig. 1) shows the two rings to adopt a perfect chair conformation as indicated by Cremer & Pople (1975) puckering parameters Q(T)= 0.573 (3)Å and spherical polar angle θ = 176.9 (3)° with φ = 281 (8)° for the first ring (C1,C6··· C10) and Q(T)= 0.571 (3)Å with a spherical polar angle θ = 173.0 (3)° and φ = 144 (3)° for the second ring (C1, C6···C10) (Cremer and Pople,1975). Molecules are linked by intermolecular O—H···O hydrogen bonds (Table 1, Figure 2) to chains running parallel to the *a* axis.

### Experimental

To 1 g (4 mmol) of 2-(4a,8-Dimethyl-1,2,3,4,4a,5,6,7-octahydro- naphthalen-2-yl)- acrylic acid methyl ester dissolved in 40 ml of dichloromethane was added one equivalent of *m*-chloroperbenzoic acid at 70%. The reaction mixture was stirred at room temperature for 3 h, then treated three times with a solution of sodium bisulfite at 10%. The organic layer was then washed with distilled water three times until neutralization, dried over sodium sulfate, filtered and concentrated under reduced pressure. The residue obtained was chromatographed on silica gel eluting with hexane/ ethyl acetate (98/2) to give quantitatively the corresponding epoxide. 500 mg (1,89 mmol) of this epoxyde is dissolved with 5% of Para-toluene sulfonic acid (APTS) in 20 ml of dichloromethane. The reaction mixture was left stirring for a period of half an hour and then treated with 10 ml of a solution of sodium bicarbonate to 10%. The organic layer was dried filtered and concentrated under reduced pressure. Chromatography on silica gel, eluting with hexane/ethyl acetate (98/2) of the residue obtained, allowed us to obtain 250 mg (94.5 mmol) of the title compound which was recrystallized in dichloromethane.

### Refinement

All H atoms were fixed geometrically and treated as riding with O—H = 0.82Å, C—H = 0.93 Å(aromatic), 0.96Å (methyl), 0.97 Å (methylene), 0.98Å (methine) with *U*_iso_(H) = 1.2U_eq_ (C_aromatic_, C_methylene_, C_methine_) or *U*_iso_(H) = 1.5*U*_eq_ (O, C_methyl_). In the absence of significant anomalous scattering, the absolute configuration could not be   determined and thus Friedel pairs were merged and any references to the Flack parameter were removed.

### Crystal data

**Table:**

C_16_H_24_O_3_	*F*(000) = 576
*M**_r_* = 264.35	*D*_x_ = 1.208 Mg m^−^^3^
Orthorhombic, *P*2_1_2_1_2_1_	Mo *K*α radiation, λ = 0.71073 Å
Hall symbol: P 2ac 2ab	Cell parameters from 6881 reflections
*a* = 6.0666 (5) Å	θ = 3.5–26.4°
*b* = 10.0900 (9) Å	µ = 0.08 mm^−^^1^
*c* = 23.747 (2) Å	*T* = 296 K
*V* = 1453.6 (2) Å^3^	Prism, colourless
*Z* = 4	0.65 × 0.45 × 0.26 mm

### Data collection

**Table:**

Bruker APEXII CCD diffractometer	1202 reflections with *I* > 2σ(*I*)
Radiation source: fine-focus sealed tube	*R*_int_ = 0.050
graphite	θ_max_ = 26.4°, θ_min_ = 3.5°
φ and ω scans	*h* = −7→7
6831 measured reflections	*k* = −11→12
1745 independent reflections	*l* = −29→29

### Refinement

**Table:**

Refinement on *F*^2^	Secondary atom site location: difference Fourier map
Least-squares matrix: full	Hydrogen site location: inferred from neighbouring sites
*R*[*F*^2^ > 2σ(*F*^2^)] = 0.046	H-atom parameters constrained
*wR*(*F*^2^) = 0.114	*w* = 1/[σ^2^(*F*_o_^2^) + (0.056*P*)^2^ + 0.0754*P*] where *P* = (*F*_o_^2^ + 2*F*_c_^2^)/3
*S* = 1.04	(Δ/σ)_max_ = 0.001
1745 reflections	Δρ_max_ = 0.18 e Å^−^^3^
176 parameters	Δρ_min_ = −0.19 e Å^−^^3^
0 restraints	Extinction correction: *SHELXL97* (Sheldrick, 2008), Fc^*^=kFc[1+0.001xFc^2^λ^3^/sin(2θ)]^-1/4^
Primary atom site location: structure-invariant direct methods	Extinction coefficient: 0.009 (2)

### Special details

**Table:**

Geometry. All s.u.'s (except the s.u. in the dihedral angle between two l.s. planes) are estimated using the full covariance matrix. The cell s.u.'s are taken into account individually in the estimation of s.u.'s in distances, angles and torsion angles; correlations between s.u.'s in cell parameters are only used when they are defined by crystal symmetry. An approximate (isotropic) treatment of cell s.u.'s is used for estimating s.u.'s involving l.s. planes.
Refinement. Refinement of *F*^2^ against ALL reflections. The weighted *R*-factor *wR* and goodness of fit *S* are based on *F*^2^, conventional *R*-factors *R* are based on *F*, with *F* set to zero for negative *F*^2^. The threshold expression of *F*^2^ > 2σ(*F*^2^) is used only for calculating *R*-factors(gt) *etc*. and is not relevant to the choice of reflections for refinement. *R*-factors based on *F*^2^ are statistically about twice as large as those based on *F*, and *R*- factors based on ALL data will be even larger.

### Fractional atomic coordinates and isotropic or equivalent isotropic displacement parameters (Å^2^)

**Table:**

	*x*	*y*	*z*	*U*_iso_*/*U*_eq_	
C1	0.1942 (5)	0.9387 (3)	0.90963 (10)	0.0356 (7)	
H1A	0.0601	0.9883	0.9026	0.043*	
H1B	0.1598	0.8451	0.9064	0.043*	
C2	0.2754 (5)	0.9680 (3)	0.96934 (11)	0.0383 (7)	
H2	0.4089	0.9153	0.9756	0.046*	
C3	0.3401 (6)	1.1152 (3)	0.97421 (12)	0.0471 (8)	
H3A	0.2093	1.1697	0.9702	0.056*	
H3B	0.4019	1.1315	1.0112	0.056*	
C4	0.5077 (5)	1.1547 (3)	0.92942 (12)	0.0443 (8)	
H4A	0.6448	1.1082	0.9365	0.053*	
H4B	0.5369	1.2489	0.9326	0.053*	
C4A	0.4312 (5)	1.1240 (3)	0.86892 (11)	0.0347 (7)	
C5	0.6221 (5)	1.1502 (3)	0.82769 (12)	0.0449 (8)	
H5A	0.6604	1.2435	0.8291	0.054*	
H5B	0.7500	1.1000	0.8397	0.054*	
C6	0.5660 (6)	1.1129 (4)	0.76711 (13)	0.0538 (9)	
H6A	0.6967	1.1222	0.7439	0.065*	
H6B	0.4549	1.1733	0.7528	0.065*	
C7	0.4804 (5)	0.9707 (3)	0.76277 (12)	0.0458 (8)	
H7A	0.4242	0.9554	0.7251	0.055*	
H7B	0.6012	0.9094	0.7691	0.055*	
C8	0.3005 (5)	0.9442 (3)	0.80496 (11)	0.0364 (7)	
C8A	0.3675 (4)	0.9758 (3)	0.86540 (11)	0.0323 (7)	
C9	0.1081 (5)	0.9271 (3)	1.01354 (12)	0.0417 (8)	
C10	0.1797 (6)	0.9201 (3)	1.07371 (12)	0.0438 (8)	
C11	0.4791 (6)	0.9357 (4)	1.13715 (12)	0.0622 (10)	
H11A	0.4307	1.0140	1.1565	0.093*	
H11B	0.6373	0.9337	1.1364	0.093*	
H11C	0.4251	0.8586	1.1564	0.093*	
C12	0.2369 (5)	1.2136 (3)	0.85384 (14)	0.0507 (9)	
H12A	0.2844	1.3044	0.8537	0.076*	
H12B	0.1217	1.2024	0.8811	0.076*	
H12C	0.1824	1.1904	0.8172	0.076*	
C13	0.1041 (5)	0.8994 (3)	0.79041 (12)	0.0480 (8)	
H13A	0.0723	0.8829	0.7527	0.058*	
H13B	−0.0025	0.8843	0.8178	0.058*	
C15	−0.0984 (6)	0.8966 (4)	1.00336 (14)	0.0586 (10)	
H15A	−0.1910	0.8724	1.0328	0.070*	
H15B	−0.1520	0.8992	0.9667	0.070*	
O1	0.5665 (3)	0.9020 (2)	0.87737 (8)	0.0406 (5)	
H1	0.5425	0.8225	0.8734	0.061*	
O2	0.3955 (4)	0.9371 (2)	1.08027 (8)	0.0514 (6)	
O3	0.0589 (4)	0.8972 (3)	1.11270 (9)	0.0684 (8)	

### Atomic displacement parameters (Å^2^)

**Table:**

	*U*^11^	*U*^22^	*U*^33^	*U*^12^	*U*^13^	*U*^23^
C1	0.0353 (17)	0.0361 (17)	0.0354 (15)	−0.0003 (14)	0.0008 (12)	0.0014 (14)
C2	0.0363 (17)	0.0434 (19)	0.0352 (15)	−0.0013 (15)	0.0007 (12)	0.0009 (14)
C3	0.056 (2)	0.047 (2)	0.0380 (15)	−0.0083 (17)	0.0031 (15)	−0.0083 (15)
C4	0.049 (2)	0.0409 (18)	0.0430 (17)	−0.0123 (16)	−0.0021 (15)	−0.0041 (14)
C4A	0.0386 (17)	0.0309 (16)	0.0344 (13)	−0.0028 (13)	−0.0020 (13)	0.0018 (12)
C5	0.046 (2)	0.0416 (19)	0.0469 (17)	−0.0073 (16)	0.0046 (15)	0.0040 (15)
C6	0.060 (2)	0.057 (2)	0.0444 (16)	−0.0056 (19)	0.0084 (17)	0.0056 (17)
C7	0.055 (2)	0.051 (2)	0.0320 (14)	−0.0024 (17)	0.0040 (14)	−0.0020 (15)
C8	0.0417 (18)	0.0327 (17)	0.0348 (14)	0.0041 (15)	−0.0043 (13)	−0.0010 (14)
C8A	0.0308 (17)	0.0316 (16)	0.0347 (14)	0.0022 (12)	−0.0012 (12)	0.0023 (13)
C9	0.0396 (19)	0.047 (2)	0.0387 (14)	−0.0005 (16)	0.0036 (13)	−0.0008 (15)
C10	0.047 (2)	0.046 (2)	0.0386 (16)	−0.0012 (16)	0.0038 (15)	0.0002 (15)
C11	0.062 (2)	0.086 (3)	0.0396 (16)	0.001 (2)	−0.0049 (15)	−0.0004 (18)
C12	0.055 (2)	0.0378 (19)	0.059 (2)	0.0032 (16)	−0.0014 (16)	0.0036 (17)
C13	0.049 (2)	0.056 (2)	0.0389 (15)	−0.0014 (18)	−0.0085 (14)	−0.0026 (16)
C15	0.044 (2)	0.080 (3)	0.0513 (19)	−0.008 (2)	0.0048 (16)	0.0058 (19)
O1	0.0377 (12)	0.0393 (12)	0.0447 (11)	0.0068 (10)	−0.0038 (9)	0.0005 (11)
O2	0.0436 (14)	0.0750 (17)	0.0354 (10)	−0.0031 (13)	0.0001 (9)	0.0007 (12)
O3	0.0595 (16)	0.103 (2)	0.0431 (12)	−0.0137 (16)	0.0125 (11)	0.0082 (13)

### Geometric parameters (Å, °)

**Table:**

C1—C2	1.530 (4)	C7—C8	1.506 (4)
C1—C8A	1.533 (4)	C7—H7A	0.9700
C1—H1A	0.9700	C7—H7B	0.9700
C1—H1B	0.9700	C8—C13	1.320 (4)
C2—C9	1.518 (4)	C8—C8A	1.525 (4)
C2—C3	1.541 (4)	C8A—O1	1.446 (3)
C2—H2	0.9800	C9—C15	1.312 (5)
C3—C4	1.524 (4)	C9—C10	1.495 (4)
C3—H3A	0.9700	C10—O3	1.203 (4)
C3—H3B	0.9700	C10—O2	1.330 (4)
C4—C4A	1.541 (4)	C11—O2	1.443 (3)
C4—H4A	0.9700	C11—H11A	0.9600
C4—H4B	0.9700	C11—H11B	0.9600
C4A—C12	1.528 (4)	C11—H11C	0.9600
C4A—C5	1.539 (4)	C12—H12A	0.9600
C4A—C8A	1.547 (4)	C12—H12B	0.9600
C5—C6	1.526 (4)	C12—H12C	0.9600
C5—H5A	0.9700	C13—H13A	0.9300
C5—H5B	0.9700	C13—H13B	0.9300
C6—C7	1.530 (5)	C15—H15A	0.9300
C6—H6A	0.9700	C15—H15B	0.9300
C6—H6B	0.9700	O1—H1	0.8200
			
C2—C1—C8A	111.5 (2)	C8—C7—C6	111.6 (3)
C2—C1—H1A	109.3	C8—C7—H7A	109.3
C8A—C1—H1A	109.3	C6—C7—H7A	109.3
C2—C1—H1B	109.3	C8—C7—H7B	109.3
C8A—C1—H1B	109.3	C6—C7—H7B	109.3
H1A—C1—H1B	108.0	H7A—C7—H7B	108.0
C9—C2—C1	111.9 (2)	C13—C8—C7	122.7 (3)
C9—C2—C3	112.4 (2)	C13—C8—C8A	124.0 (3)
C1—C2—C3	109.8 (2)	C7—C8—C8A	113.3 (2)
C9—C2—H2	107.5	O1—C8A—C8	107.5 (2)
C1—C2—H2	107.5	O1—C8A—C1	108.2 (2)
C3—C2—H2	107.5	C8—C8A—C1	114.2 (2)
C4—C3—C2	111.7 (2)	O1—C8A—C4A	106.2 (2)
C4—C3—H3A	109.3	C8—C8A—C4A	108.6 (2)
C2—C3—H3A	109.3	C1—C8A—C4A	111.7 (2)
C4—C3—H3B	109.3	C15—C9—C10	116.3 (3)
C2—C3—H3B	109.3	C15—C9—C2	125.1 (3)
H3A—C3—H3B	107.9	C10—C9—C2	118.7 (3)
C3—C4—C4A	113.4 (3)	O3—C10—O2	122.3 (3)
C3—C4—H4A	108.9	O3—C10—C9	124.6 (3)
C4A—C4—H4A	108.9	O2—C10—C9	113.1 (3)
C3—C4—H4B	108.9	O2—C11—H11A	109.5
C4A—C4—H4B	108.9	O2—C11—H11B	109.5
H4A—C4—H4B	107.7	H11A—C11—H11B	109.5
C12—C4A—C5	109.2 (2)	O2—C11—H11C	109.5
C12—C4A—C4	109.4 (2)	H11A—C11—H11C	109.5
C5—C4A—C4	109.4 (2)	H11B—C11—H11C	109.5
C12—C4A—C8A	111.5 (2)	C4A—C12—H12A	109.5
C5—C4A—C8A	108.6 (2)	C4A—C12—H12B	109.5
C4—C4A—C8A	108.6 (2)	H12A—C12—H12B	109.5
C6—C5—C4A	112.9 (3)	C4A—C12—H12C	109.5
C6—C5—H5A	109.0	H12A—C12—H12C	109.5
C4A—C5—H5A	109.0	H12B—C12—H12C	109.5
C6—C5—H5B	109.0	C8—C13—H13A	120.0
C4A—C5—H5B	109.0	C8—C13—H13B	120.0
H5A—C5—H5B	107.8	H13A—C13—H13B	120.0
C5—C6—C7	111.8 (3)	C9—C15—H15A	120.0
C5—C6—H6A	109.3	C9—C15—H15B	120.0
C7—C6—H6A	109.3	H15A—C15—H15B	120.0
C5—C6—H6B	109.3	C8A—O1—H1	109.5
C7—C6—H6B	109.3	C10—O2—C11	117.1 (2)
H6A—C6—H6B	107.9		

### Hydrogen-bond geometry (Å, °)

**Table:**

*D*—H···*A*	*D*—H	H···*A*	*D*···*A*	*D*—H···*A*
O1—H1···O3^i^	0.82	2.24	3.033 (4)	161

Symmetry codes: (i) *x*+1/2, −*y*+3/2, −*z*+2.
